# Molecular characterization, biofilm analysis and experimental biofouling study of *Fusarium *isolates from recent cases of fungal keratitis in New York State

**DOI:** 10.1186/1471-2415-7-1

**Published:** 2007-01-30

**Authors:** Madhu Dyavaiah, Rama Ramani, David S Chu, David C Ritterband, Mahendra K Shah, William A Samsonoff, Sudha Chaturvedi, Vishnu Chaturvedi

**Affiliations:** 1 Mycology Laboratory, Wadsworth Center, New York State Department of Health, 120 New Scotland Avenue, Albany, NY 12208-2002, USA; 2 Department of Biomedical Sciences, School of Public Health, SUNY Albany, 120 New Scotland Avenue, Albany, NY 12208-2002, USA; 3 Division of Cornea and Refractive Surgery, Institute of Ophthalmology and Visual Science, New Jersey Medical School, Newark, NJ 07101-1709, USA; 4 Department of Ophthalmology, New York Eye and Ear Infirmary, New York, NY 10003, USA; 5 Department of Pathology and Laboratory Medicine, New York Eye and Ear Infirmary, New York, NY 10003, USA; 6 Electron Microscopy Core, Wadsworth Center, New York State Department of Health, Empire State Plaza, Albany, NY 12201-0509, USA

## Abstract

**Background:**

To characterize *Fusarium *isolates from recent cases of fungal keratitis in contact lens wearers, and to investigate fungal association with MoistureLoc solution.

**Methods:**

We studied six fungal isolates from recent cases of keratitis in New York State. The isolates were characterized by nucleotide sequencing and phylogenetic analyses of multiple genes, and then typed using minisatellite and microsatellite probes. Experimental fungal biofilm formation was tested by standard methods. MoistureLoc solutions were tested in biofouling studies for their efficacy in elimination of *Fusarium *contamination.

**Results:**

*Fusarium solani *– corneal ulcers (2 isolates), lens case (1 isolate), and *F. oxysporum *– corneal ulcer (1 isolate), eye (1 isolate), were recovered from five patients. An opened bottle of MoistureLoc solution provided by a patient also yielded *F. solani*. Two distinct genotypes of *F. solani *as well as of *F. oxysporum *were present in the isolated strains. Remarkably, *F. solani *strains from the lens case and lens solution in one instance were similar, based on phylogenetic analyses and molecular typing. The solution isolate of *F. solani *formed biofilm on contact lenses in control conditions, but not when co-incubated with MoistureLoc solution. Both freshly opened and 3-month old MoistureLoc solutions effectively killed *F. solani *and *F. oxysporum*, when fungal contamination was simulated under recommended lens treatment regimen (4-hr). However, simulation of inappropriate use (15 – 60 min) led to the recovery of less than 1% of original inoculum of *F. solani *or *F. oxysporum*.

**Conclusion:**

Temporary survival of *F. solani *and *F. oxysporum *in MoistureLoc suggested that improper lens cleaning regimen could be a possible contributing factor in recent infections.

## Background

Fungal keratitis due to filamentous fungi is quite common in tropical parts of the world and the southeastern United States [1-3]. Farmers and workers in agro-industries are most at risk for these infections, due to occupation-related corneal abrasions and subsequent fungal infections. In temperate regions, fungal keratitis is most commonly caused by the yeast *Candida albicans*, although mold keratitis due to *Fusarium *is being increasingly recognized [4-6]. Contact lens wearers have an elevated risk for fungal keratitis, but the incidence of *Fusarium *keratitis is quite rare among this group of patients [7-9]. There has been no report until the beginning of 2006, on the association of multipurpose contact lens solutions with fungal keratitis.

An upsurge in *Fusarium *keratitis among contact lens wearers was first noticed in 2005 by public health authorities in Singapore. Subsequently, 66 patients were identified from March 2005-February 2006; the characteristics in common among these patients were the use of ReNu with MoistureLoc solution and poor lens hygiene [10]. Two recent reports described a noticeable increase in *Fusarium *keratitis in contact lens wearers in the US, in the San Francisco Bay Area and in southern Florida [11,12]. A more comprehensive report has documented 176 cases of *Fusarium *keratitis in 164 patients from various regions in the US [13]. The investigators found significant use of MoistureLoc solution among 45 case patients in their series. Interestingly, they did not recover any *Fusarium *isolate through extensive sampling of lens solutions from the patients, or from environmental sampling of the plant where the solution was manufactured. Thus, the underlying causes of this perplexing outbreak remain elusive. We investigated recent fungal keratitis cases from New York State including the first case in which *Fusarium *was isolated from MoistureLoc solution being used by a patient. We also carried out experimental studies with *Fusarium *isolates in attempts to understand why these infections were associated with the use of MoistureLoc solution.

## Methods

### Mycology

Five *Fusarium *isolates, recovered from patients' corneal ulcers, contact lenses, or contact lens cases by hospital laboratories in New York and New Jersey, were forwarded to the Mycology Laboratory at the Wadsworth Center (Table 1). These isolates were studied in detail for their colony and microscopic characteristics [14,15]. An opened container of MoistureLoc solution being used by a patient for approximately three-months was also submitted for evaluation. An aliquot of this solution was directly plated on Sabouraud's dextrose agar supplemented with antibiotics (chloramphenicol 25 μg/ml, gentamicin 40 μg/ml) for fungal recovery, and the solution was centrifuged at 5000 rpm for 15 min to recover fungi from the pellet and the supernatant, by plating on culture medium. Additional unopened bottles of MoistureLoc solution were procured from local pharmacies in Albany and New York City before the global recall of this product by the manufacturer. These solutions were processed for fungal testing at the Wadsworth Center and the New York Eye and Ear Infirmary.

**Table 1:** *Fusarium *species isolated from recent cases of keratitis

Isolate No.	Source	Identification
23-06	Corneal ulcer	*F. solani*
24-06	Corneal ulcer/contact lens	*F. solani*
158-06	Eye	*F. oxysporum*
159-06	Corneal ulcer	*F. oxysporum*
237-06	Lens case	*F. solani*
238-06	MoistureLoc bottle	*F. solani*

### Molecular characterization

Specific identifications and typing of *Fusarium *isolates were done by PCR and nucleotide sequencing. Fungal DNA extraction involved grinding mycelia in a pestle and mortar under liquid nitrogen, suspending the slurry in lysis buffer (200 mM Tris-HCl, pH 8.5, 250 mM NaCl, 25 mM EDTA, 0.5%SDS), extraction with phenol: chloroform: isoamyl alcohol, and ethanol precipitation [16]. Fungal ribosomal DNA internal transcribed spacers 1 and 2 (*ITS1, ITS2*) and nuclear 28S rRNA [17], and *Fusarium-*specific partial β-tubulin and elongation factor (*EF-1α*) genes were amplified with published primers [18]. Nucleotide sequencing of the PCR amplicons was done on both strands according to the standard methods [19]. Nucleotide sequences were manually edited and compared against the NCBI database and *Fusarium *database at the Pennsylvania State University [20]. These sequences have been deposited in the GenBank under Accession Numbers DQ852626 – DQ852630 and DQ813505 – DQ813508. Percentage of nucleotide identity among various genes was compared by ClustalW (v1.4) multiple alignment, using MacVector 7.1.1 software (Accelrys, San Diego, CA). Phylogenetic analyses of nucleotide sequences were done with the PAUP v4 program using a bootstrap method with a Neighbor-Joining or Maximum Parsimony search [21].

Molecular typing of *Fusarium *isolates was carried out using two random probes: a 15-bp minisatellite probe from M13 bacteriophage [22] and a simple DNA repeat (GACA)_4 _probe [23]. These probes were chosen given that they have been widely used to discriminate between related strains of a variety of pathogenic microorganisms. Three laboratory isolates- *F. oxysporum *163-05 (corneal ulcer), *F. oxysporum *974-05 (finger nail) and *F. solani *1064-05 (corneal ulcer), unrelated to this outbreak, were used as outliers to check robustness of the two genotyping methods. Single M13 (5' GAGGGTGGCGGTTCT 3') and (GACA)_4 _primers were used in PCR reaction. PCR reaction volume of 50 μl included 5 μl of 10 × PCR buffer with 15 mM MgCl_2_, 3.0 μl dNTP mix (10 μmol/L each), 30 ng primer, 2.5 U Taq DNA polymerase (Perkin Elmer, Foster City, CA, USA). Initial denaturation was at 94°C for 20 sec, followed by 35 cycles of denaturation at 94°C for 20 sec, annealing at 50°C for 1 min, amplification at 72°C for 20 sec and final extension 72°C for 4 min, in a GenAmp PCR System 2400 (Perkin Elmer). PCR products were concentrated to 20 μl and resolved by electrophoresis on 1.4% agarose gels in Tris-borate-EDTA (TBE) buffer, and were detected by ethidium bromide staining [24].

### Biofilm analysis

Microbial biofilms are an important potential source of infectious propagules in keratitis [25,26]. Therefore, the ability of the *Fusarium *isolates to form biofilms in the laboratory was tested with etafilcon A contact lenses (ACUVUE^®^, Johnson & Johnson Vision Care, Inc.) using a published procedure [25]. Briefly, *F. solani *238-06 was grown on Sabouraud's dextrose agar slants for 5 days, and the conidia harvested in sterile distilled water. Fungal inoculum comprised of conidial suspension adjusted to 1 × 10^4 ^cells/ml with a hemacytometer. A balanced salt solution (0.49 g NaCl, 0.075 g KCl, 0.048 g CaCl_2_, 0.03 g MgCl_2_, 0.39 g sodium acetate {CH_3_COONa}, 0.17 g sodium citrate {HOC(COONa)(CH2COONa)2}, dissolved in 100 ml deionized water, and filter sterilized) was used to promote biofilm formation [25]. Five ml balanced salt solution aliquots were transferred to 25-ml flasks. Half of the flasks received sterile, fresh, ACUVUE contact lens and 10 μl of the fungal inoculum. The other flasks received contact lens, fungal inoculum and 1.0 ml MoistureLoc. The negative control was balanced salt solution with contact lens but no fungal inoculum. The flasks were incubated for 48 h at 25°C on a rotator (180 rpm). Some lenses from flasks with balanced salt solution and fungal inoculum but no MoistureLoc were removed at the end of incubation, treated with MoistureLoc for 4 h and processed for scanning electron microscopy (SEM). The contact lenses were fixed in 2% glutaraldehyde for 1 h, and dehydrated in 25–100% ethanol. Dehydrated lenses were critical point dried, coated with gold particles and examined in SEM as described previously [19].

### Experimental biofouling

A previous report indicated that the time elapsed since opening and the storage conditions significantly affected the efficacy of the multipurpose contact lens solutions [27]. We designed a biofouling study based on earlier publications to test for efficacy of MoistureLoc solutions against *Fusarium *isolates [28,29]. MoistureLoc solutions, purchased from local pharmacies before the global recall of this product, were inoculated with freshly grown *Fusarium *to test for survival and growth. Both sterile glass test tubes (20 × 150 mm) and commercial lens cases (Sight Savers^®^, Bausch & Lomb, Inc.) were used in these experiments. *F. solani *238-06 and *F. oxysporum *158-06, from 4–5 day old slants of Sabouraud's dextrose agar, were suspended in sterile distilled water. The suspension comprised mostly of conidia with rare hyphal fragments. The conidia were counted using a hemacytometer, and adjusted to a final density of 1 × 10^4 ^– 10^7 ^cells/ml. Ten microliters of these suspensions were used to inoculate 1.0 ml of MoistureLoc. The mix was incubated unstirred at 23° – 25°C for various intervals, and 100 μl aliquots were spread on Sabouraud's dextrose agar plates after 15, 30, 60 and 240 min. The positive control included fungal inoculum suspended in sterile water without MoistureLoc. The inoculated plates were incubated at 25°C for 48 – 72 h and fungal colonies counted and photographed. [28,29]. The biofouling experiments were repeated 5 times.

## Results

### Mycology

**Figure 1:**
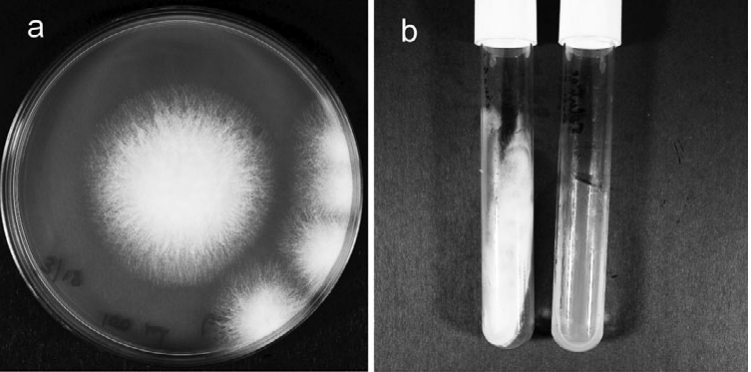
**Recovery of *Fusarium *from lens solution**. (a) *Fusarium solani *colonies recovered from 100 μl of MoistureLoc cleaning solution provided by a patient (b) *F. solani *colonies on Sabouraud's dextrose agar slants inoculated with supernatant (left); in contrast no colonies were recovered from the pellet (right) from the same MoistureLoc solution.

MoistureLoc solution, provided by a patient, was positive for *Fusarium *species, and quantification of fungal load revealed approximately 50 colonies/ml (figure 1a). Furthermore, the supernatant, and not the pellet from the contaminated solution yielded the fungus (figure 1b). The finding was consistent with moderate recovery of fungal colonies from this solution. Characterization of the morphology and spores of the MoistureLoc isolate (238-06) showed it to most closely resemble *F. solani*. Mycological investigations of five patient isolates also showed them to be either *F. solani *or *F. oxysporum *(Table 1).

### Molecular characterization

**Figure 2:**
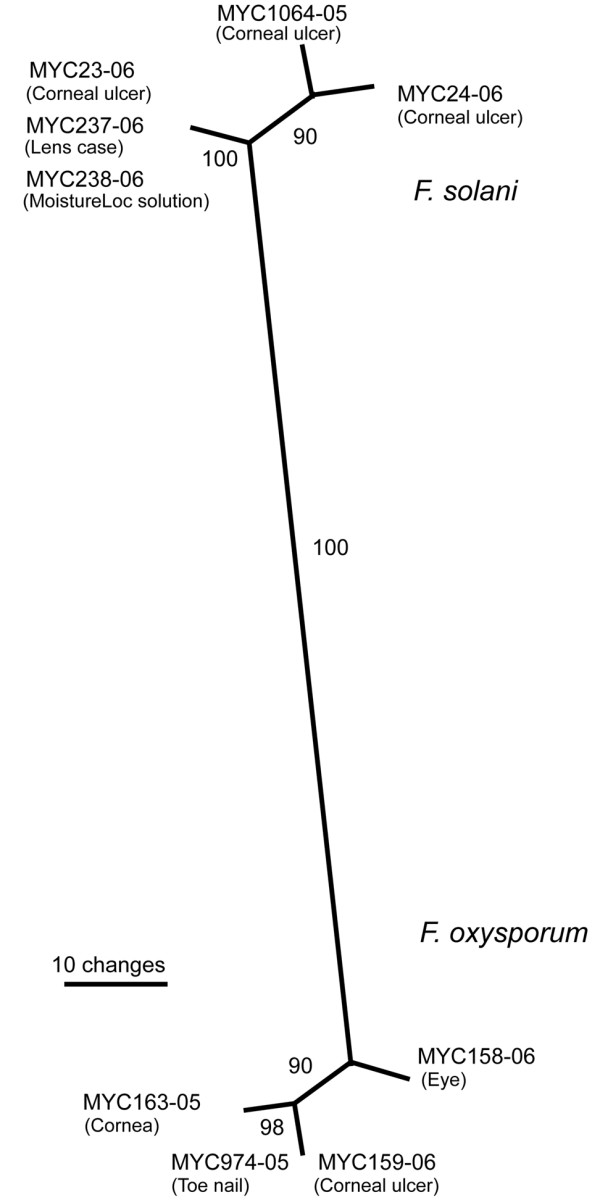
**Unrooted parsimony phylogram of *Fusarium EF-1α *gene sequences**. Nucleotide sequences of *EF-1α *gene were compared by ClustalW multiple alignment using MacVector 7.1.1 software (Accelrys, San Diego, CA). Phylogenetic analyses were done with the PAUP v4 program using a bootstrap method with a Neighbor-Joining or Maximum Parsimony search [21]. The numbers on the branches indicate percent bootstrap values, based on 1000 replicates. Three distinct clades were seen in both the *F. oxysporum *and the *F. solani *strains. The six strains from our investigation came from five different patients: 23-06 (corneal ulcer), 24-06 (contact lens), 158-06 (right eye), 159-06 (corneal ulcer left eye), 237-06 and 238-06 (contact lens case swab and contact lens cleaning solution from the same patient). Three outlier controls were *F. oxysporum *(163-05) and *F. solani *(1064-05), previously isolated from unrelated keratitis cases, and *F. oxysporum *(974-05) isolated from finger nail.

**Figure 3:**
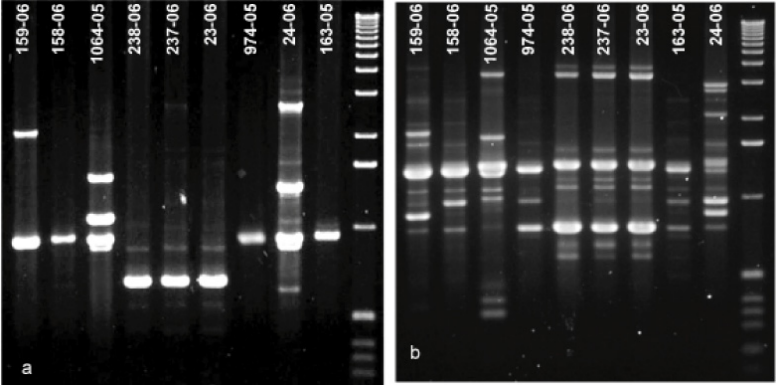
**Molecular typing of *Fusarium *isolates**. Molecular typing with (a) minisatellite probe {M13}, and (b) repeat DNA probe {GACA}_4 _showed distinct genotypes among *Fusarium *isolates. *F. solani *strain 24-06 genotype was easily separated from the other three strains (23-06, 237-06, and 238-06). *F. solani *from contact lens case (237-06) and from MoistureLoc solution (238-06) showed similar genotypes, by the two probes. In contrast, *F. oxysporum *strains 158-06 and 159-06 yielded two different genotypes. The control *F. oxysporum *(163-05, 974-05) and *F. solani *(1064-05) genotypes were distinct. The lane assignments for 163-05, 974-05, and 24-06 are different in panels (a) and (b).

Total of five genes including *ITS1*, *ITS2*, 28S rRNA, β-Tubulin, and *EF-1α *were used for confirmation of *Fusarium *species (GenBank Accession Numbers DQ852626 – DQ852630 and DQ813505 – DQ813508). Of these genes, sequences from *ITS1*, *ITS2*, and *EF-1α *possessed enough polymorphism, and therefore, were excellent marker with 99–100% accuracy for the identification of *Fusarium *species to be either *F. solani *or *F. oxysporum *species complex in the NCBI and *Fusarium *databases (hereafter referred as *F. solani *and *F. oxysporum*). On the contrary, sequences from 28S and β-tubulin genes were highly conserved and therefore, these were only of limited use in further delineation of *Fusarium *species. Multiple alignments of *EF-1α *sequences of *Fusarium *species revealed significant polymorphism between *F. solani *and *F. oxysporum *with 68–73% identity. Within *F. solani *species, three strains 23-06 (DQ813507), 237-06 (DQ813506), and 238-06 (DQ813505) exhibited 100% sequence identity and showed 92% and 84% identities with strains 1064-05 (DQ852626), and 24-06 (DQ813508), respectively. Similarly, within *F. oxysporum *species, four strains namely 158-05 (DQ852627), 159-06 (DQ852628.), 163-05 (DQ852629), and 974-05 (DQ852630) exhibited small variation in their *EF-1α *sequences with 93–95% identity. Phylogenetic analysis of various gene fragments amplified from these isolates showed distinct clades of *F. solani *and *F. oxysporum *in the test group. Two unrelated strains of *F. oxysporum *(163-05, 974-05) and one *F. solani *strain (1064-05), used as controls in these studies, also segregated into distinct clades. A representative phylogram from *EF-1α *gene fragment is shown in figure 2. Subsequent molecular typing with M13 and (GACA)_4 _primers generated genotypic patterns that permitted distinct groupings of the test strains (figure 3). Two distinct genotypes of *F. solani *as well as of *F. oxysporum *were present in the isolated strains. *F. solani *strain 24-06 genotype was easily separated from the other three strains (23-06, 237-06, and 238-06). *F. solani *from contact lens case (237-06) and from MoistureLoc solution (238-06) showed similar genotypes, by the two probes. In contrast, *F. oxysporum *strains 158-06 and 159-06 yielded two different genotypes. The control *F. oxysporum *(163-05, 974-05) and *F. solani *(1064-05) genotypes were distinct.

### Experimental studies

**Figure 4:**
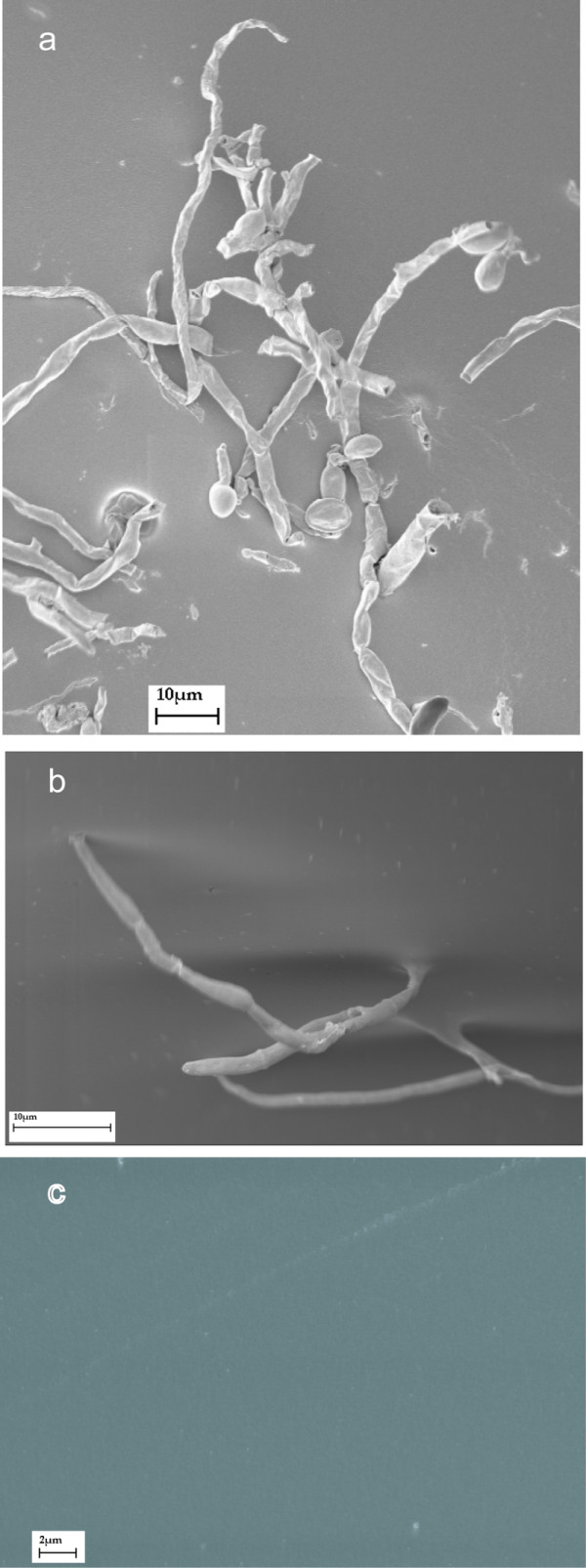
**Scanning electron micrographs of biofilm formation**. (a) *F. solani *(238-06) incubated with fresh ACUVUE lens and balanced salt solution for 48 h at 25°C on a rotator (180 rpm). (b) Close-up of the same isolate showing hyphal attachment to the lens surface (c) Disappearance of biofilm when preparation from (a) was treated with MoistureLoc for 4 h.

**Figure 5:**
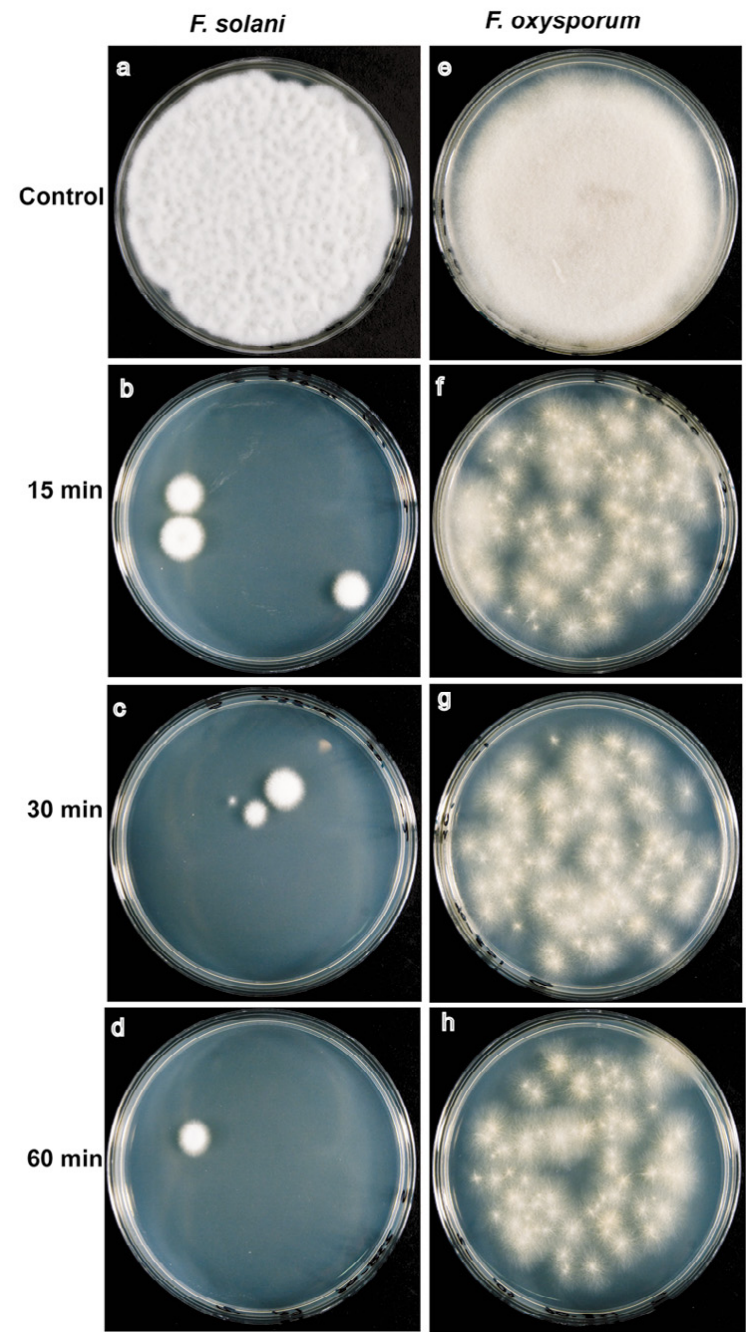
**Experimental biofouling studies with *F. solani *(238-06) and *F. oxysporum *(158-06)**. Fungal cells (1 × 10^5^) were inoculated into 1.0 ml of MoistureLoc solution, and 100-μl aliquots were plated on yeast extract-peptone-dextrose agar at intervals (a-h). Fungal colonies were recovered from MoistureLoc^® ^solutions sampled at 15, 30 and 60 min intervals (b-d) *F. solani*; (f-h) *F. oxysporum*. All solutions were sterile after the manufacturer recommended length of treatment, 4 hr (data not shown). The total numbers of colonies recovered are ten-fold of the dilution shown in these illustrations; the data is summarized in figure 6. Similar results were seen when 3-months old and freshly opened MoistureLoc solutions were compared. The experiments were repeated at least three times; a representative experiment is shown.

**Figure 6:**
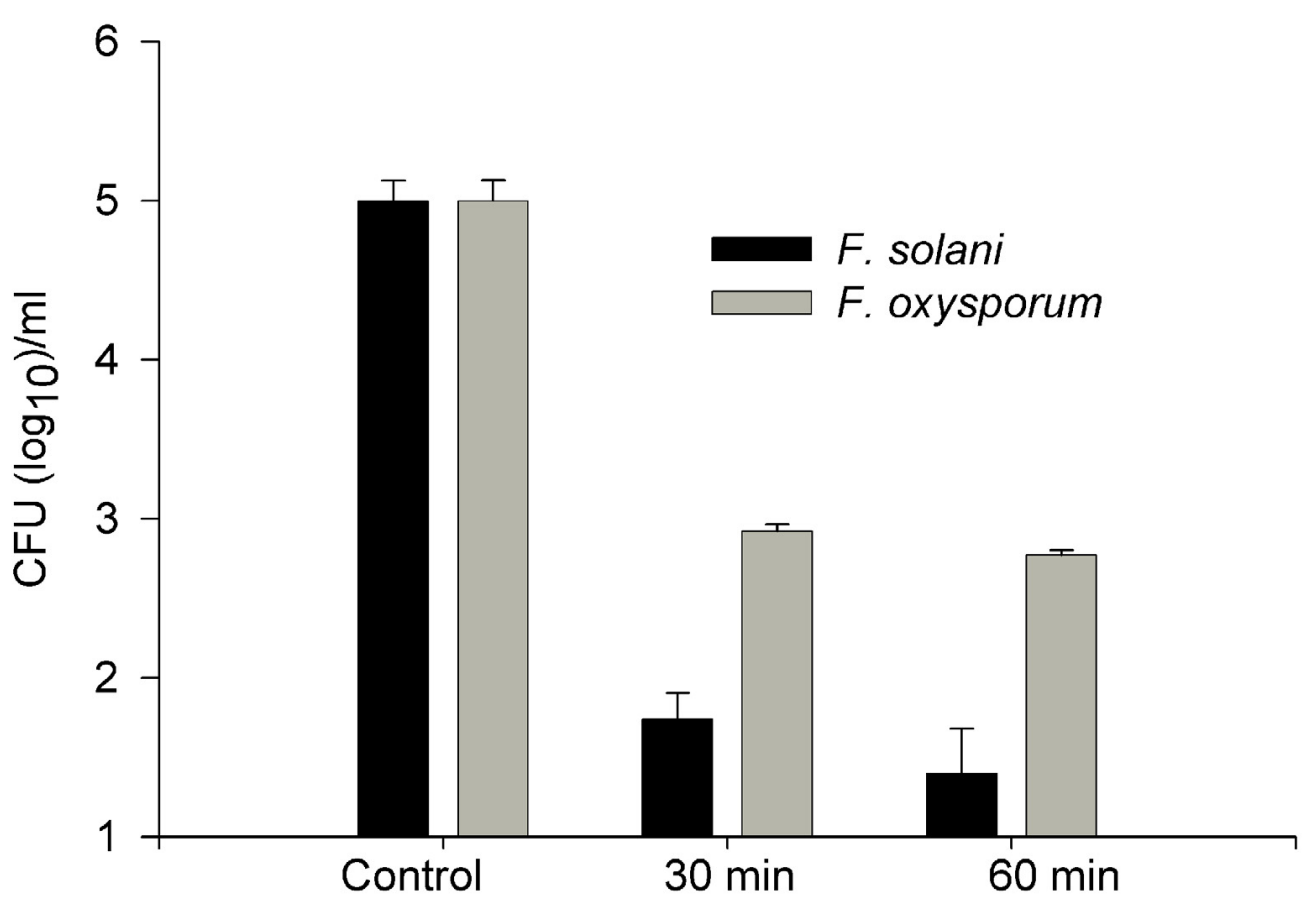
**Summary of experimental biofouling studies**. The experimental biofouling studies were carried out by introducing 1 × 10^5 ^fungal cells (colony forming units; CFU) of *F. solani *(238-06) or *F. oxysporum *(158-06) into 1.0 ml of MoistureLoc solution. The recovery of CFU after 30 and 60 minutes is shown from three representative experiments (mean ± SD). The cultures were sterile after recommended 4.0 hrs of incubation (data not shown).

In biofouling studies, *F. solani *(238-06) formed biofilms on freshly opened contact lenses incubated in balanced salt solution (figure 4). Biofilms did not form when contact lenses were incubated in balanced salt solution + MoistureLoc solution. Further, if a biofilm was allowed to form in the salt solution, the treatment with the MoistureLoc sufficed to destroy the biofilm (figure 4c). Similar negative results were obtained when patient isolates were tested for their ability to form biofilms in presence or absence of MoistureLoc (data not shown). Preliminary tests with MoistureLoc suggested that *F. solani *(238-06) did not survive or grow in shaken cultures kept for 7 days at 30°C (data not shown). Different batches of MoistureLoc solutions, procured from local pharmacies before the global recall of this product by the manufacturer, also tested sterile in our hands. MoistureLoc solutions effectively killed *F. solani *(238-06) and *F. oxysporum *(158-06) upon experimental inoculation, provided that the recommended length of treatment, 4-hr, was used. Further studies designed to simulate inappropriate cleaning revealed that approximately 25 colonies of *F. solani *(0.02%) and 700 colonies of *F. oxysporum *(0.59%) could be recovered after 60 min from the initial 100,000 colony forming units (CFU) used for inoculation of MoistureLoc solution (figures 5, 6). The remaining four test strains also yielded similar survival patterns in these experiments (details not shown). There was no appreciable difference in fungal survival rates, between glass tubes and lens cases used as container for interactions. Similarly, there was no difference in survival pattern between freshly opened MoistureLoc solution and solutions opened 3-months earlier, in these experiments.

## Discussion

Among the significant findings of our study was the recovery of similar *F. solani *strains from the MoistureLoc solution and from the contact lens case of a patient. To our knowledge, this is the first instance of matched isolations of any *Fusarium *strain connected with recent cases of fungal keratitis in contact lens wearers [10,13]. However, only one additional isolate of *F. solani*, among the four that we studied, shared this genotype. Also, two *F. oxysporum *strains in this study showed two distinct genotypes. These observations, although limited suggested that it is unlikely that common or clonal strains were the cause of the infections. This conclusion agrees with the findings of the report about the presence of multiple genotypes of *F. solani *and *F. oxysporum *in keratitis cases in contact lens wearers in the US [13]. The population make-up of a few *F. solani *isolates from Singapore and Hong Kong also showed mixed genotypes, albeit with one population predominating; the latter was common in both locales. More comprehensive studies are indicated to rule out the presence of 'hypervirulent clones' as the cause of recent keratitis cases. It could be pertinent that a number of other investigators have pointed out that clinical strains of *Fusarium *are generally a mix of genotypes of plant and environmental origin [13,20,30,31].

It was reasonable to suspect that devices such as contact lenses or lens cases are important in the etiology of these infections. In fact, the storage cases are reported to pose an independent risk factor for biofilm formation in mycotic keratitis [26]. We did not receive any relevant specimens for mycology and/or microscopy that would have enabled us to further investigate this aspect. Our limited experimental analyses indicated that both *F. solani *and *F. oxysporum *strains were capable of forming biofilms on these devices under permissive conditions, but not in presence of or after recommended treatment with MoistureLoc solution. Based on this evidence, we suggest that the possible sources of *Fusarium *in keratitis patients are unlikely to include contact lenses or lens cases when MoistureLoc solution is properly used. However, further studies are indicated to examine if contaminated lenses or lens cases could still be important sources of infections independent of lens cleaning regimen.

Our recovery of *F. solani *from MoistureLoc solution provided by a patient, taken with the previous report of a strong association between MoistureLoc solution and keratitis cases, led us to evaluate the scenarios in which the efficacy of this multipurpose solution would be compromised. We found that MoistureLoc solution completely sterilized *F. solani *and *F. oxysporum *contaminations as long as recommended 4-hr treatment regimen was followed. These observations were consistent with the conclusions of many other reports about the overall efficacy of various multipurpose solutions against fungi [28,29]. However, it has been documented that the efficacy of cleaning solutions depends on the storage temperature and the time elapsed after opening of the container [27]. Both of these variables were examined to a limited extent in our study; our experiments were carried out with freshly opened containers and with containers opened roughly 3-months earlier with storage at room temperature. Again, no appreciable differences in efficacy were noticed between fresh and aged solutions, experimentally inoculated with *F. solani *and *F. oxysporum*. The simulation of inappropriate usage of MoistureLoc solution yielded positive growth up to 60 min with *F. oxysporum *being 30-fold more resistant than *F. solani*. These results suggested that at least some occurrences of keratitis could result from temporary contamination and/or inadequate cleansing with MoistureLoc solution.

## Conclusion

Temporary survival of *F. solani *and *F. oxysporum *in MoistureLoc suggested that improper lens cleaning regimen could be a possible contributing factor in recent infections.

## Competing interests

The author(s) declare that they have no competing interests.

## Authors' contributions

**MD: **Performed molecular typing and experimental biofouling studies

**RR: **Performed mycology and biofilm studies

**DSC: **Provision of patient material, critical revision of the article

**DCR: **Provision of patient material, critical revision of the article

**MKS: **Data collection, provision of materials

**WAS: **Performed electron microscopy

**SC: **Data analysis and interpretation, critical revision of the article

**VC: **Conception and design, analysis and interpretation, writing the draft manuscript, provision of materials and resources

All authors have read and approved the final manuscript.

## Pre-publication history

The pre-publication history for this paper can be accessed here:


